# Fractions of Shen-Sui-Tong-Zhi Formula Enhance Osteogenesis *Via* Activation of β-Catenin Signaling in Growth Plate Chondrocytes

**DOI:** 10.3389/fphar.2021.711004

**Published:** 2021-09-24

**Authors:** Rui Xu, Qinghe Zeng, Chenjie Xia, Jiali Chen, Pinger Wang, Shan Zhao, Wenhua Yuan, Zhaohuan Lou, Houfu Lin, Hanting Xia, Shuaijie Lv, Taotao Xu, Peijian Tong, Mancang Gu, Hongting Jin

**Affiliations:** ^1^ Institute of Orthopedics and Traumatology, The First Affiliated Hospital of Zhejiang Chinese Medical University, Hangzhou, China; ^2^ The First College of Clinical Medicine, Zhejiang Chinese Medical University, Hangzhou, China; ^3^ Department of Orthopedic Surgery, Zhejiang Hospital of Integrated Traditional Chinese and Western Medicine, Hangzhou, China; ^4^ Department of Orthopedic Surgery, Ningbo Medical Center Lihuili Hospital, Ningbo, China; ^5^ College of Pharmaceutical Science, Zhejiang Chinese Medical University, Hangzhou, China; ^6^ Department of Orthopedic Surgery, The First Affiliated Hospital of Zhejiang Chinese Medical University, Hangzhou, China

**Keywords:** Shensuitongzhi formula, osteoporosis, osteogenesis, β-catenin signaling, transgenic mice

## Abstract

**Background:** Shen-sui-tong-zhi formula (SSTZF) has been used to treat osteoporosis for decades and shows excellent clinical efficacy. This article aims to explore the optimal anti-osteoporotic ingredient and its precise mechanisms in mice models.

**Methods:** In this study, we first screened the optimal anti-osteoporosis fraction of SSTZF extract *in vivo*, and then further explored the mechanism of its effects both *in vivo* and *in vitro*. Ten-week-old female C57BL/6J mice were administrated with each fraction of SSTZF. At 10 weeks after ovariectomy (OVX), femurs were collected for tissue analyses, including histology, micro-CT, biomechanical tests, and immunohistochemistry for ALP, FABP4, and β-catenin. Additionally, we also evaluated the mRNA expression level of ALP and FABP4 and the protein expression level of β-catenin after being treated with SSTZF extract in C_3_H_10_T1/2 cells. Moreover, we investigated the anti-osteoporosis effect of SSTZF extract on mice with *β-catenin* conditional knockout in growth plate chondrocytes (*β-catenin* ^*Gli1ER*^ mice) through μCT, histology, and immunohistochemistry analyzes.

**Results:** At 10 weeks after treatment, osteoporosis-like phenotype were significantly ameliorated in SSTZF n-butanol extract (SSTZF-NB) group mice, as indicated by increased trabecular bone area and ALP content, and decreased lipid droplet area and FABP4 content. No such improvements were observed after being treated with other extracts, demonstrating that SSTZF-NB is the optimal anti-osteoporosis fraction. Additionally, the elevated β-catenin was revealed in both OVX mice and C_3_H_10_T1/2 cells with SSTZF-NB administered. Furthermore, a significant osteoporosis-like phenotype was observed in *β-catenin* ^*Gli1ER*^ mice as expected. However, SSTZF-NB failed to rescue the deterioration in *β-catenin* ^*Gli1ER*^ mice, no significant re-upregulated ALP and downregulated FABP4 were observed after being treated with SSTZF-NB, demonstrating that SSTZF-NB prevents bone loss mainly via β-catenin signaling.

**Conclusion:** SSTZF-NB enhances osteogenesis mainly via activation of β-catenin signaling in growth plate chondrocytes. SSTZF-NB is the optimal anti-osteoporosis fraction of SSTZF and it can be considered a salutary alternative therapeutic option for osteoporosis.

## 1 Introduction

Osteoporosis (OP) is the most common bone disorder around the world. It is characterized by fragile bone fracture, which results from reduced bone mass and deteriorated bone microstructure (Force et al., 2018). Studies have shown that there are 10 million people with osteoporosis in the United States alone, and nearly 34 million people with low bone mass, which means they suffer from an increased risk of osteoporosis (Wu et al., 2019). With the speeding tendency of an aging society, the number of potential OP patients will continuously rise. Currently, the drug for treating OP is still limited due to unexpected side effects, like the impact on the uterus and breast (Cheng et al., 2021). At the moment when Covid-19 is rampant all over the world, it will undoubtedly bring a heavy burden and pressure to the social economy and medical systems (Mattioli et al., 2020). Thus, it is essential to explore and create more alternative therapies for OP treatment (Vandenbroucke et al., 2017). Botanical drugs and their natural extracts attract more and more attention due to the potential anti-osteoporosis effects and the fact that they are relatively safe (Zhang et al., 2016; Zhu et al., 2018).

Traditional Chinese Medicine (TCM) has been widely used for various medical purposes for centuries in East Asia. According to the “kidney dominates bone” theory in TCM, OP is the result of kidney deficiency and decreased marrow. Therefore, the therapeutic strategy should emphasize tonifying kidney and regulating the marrow. Based on the theory mentioned above, the Shen-sui-tong-zhi formula (SSTZF) was devised and has been used for treating bone-related disorders for dozens of years. SSTZF is an experiential effective recipe devised by well-known doctors of the Zhejiang School and is modified from Yougui Pills, which was first recorded in *“jin yue quan shu,”* a medical classic text written by Jingyue Zhang in the Ming Dynasty. It encompasses *Carthamus tinctorius L.* (CTL)*, Rehmannia glutinosa (Gaertn.) DC.* (RG), *Eucommia ulmoides oliver* (EUO)*, Aconitum carmichaelii debeaux* (ACD)*, Lycium barbarum L.* (LBL)*, Cornus officinalis Siebold & Zucc.* (COS), *Dioscorea oppositifolia L.* (DOL) *Glycyrrhiza glabra L.* (GGL), and *Prunus davidiana (CarriŠre) Franch* (PD), *Cinnamomum cassia (L.). J.Presl* (CCL). In the context of Yougui Pills, the combination of RG, EUO, ACD, LBL, COS, DOL, GGL, and CCL has proven efficacy for bone protection and reveals the potential of activating β-catenin signaling (Yan et al., 2018; Zhang et al., 2019). In addition, several studies have indicated that CTL and its active ingredients could promote BMSCs and differentiate into osteoblasts and show a capacity for bone protection (Kim et al., 2002; Alam et al., 2006; Cui et al., 2019). PD is included to inhibit adipogenesis (Choi et al., 1991a; Choi et al., 1991b; Jung et al., 2017). Combining osteogenic botanical drugs and anti-adipogenic botanical drugs with bone-friendly components has offered a promising alternative therapy for bone related disease, especially for OP. Our previous study indicated that SSTZF drug serum promotes osteoblast proliferation and mineralization in the β-catenin related pathway *in vitro*. However, the precise underlying mechanisms and optimal fractions remain unclear, which limits its further exploitation and promotion.

Bone is a living organ in vertebrates. In clinical practice, bone loss and fat accumulation in bone marrow were found in age-related OP (Pei and Tontonoz, 2004). Even though the mechanism of OP remains unclear, more and more evidence indicates that the unbalance between osteogenesis and adipogenesis plays an important role during the progression (Colaianni et al., 2015; Zou et al., 2020). As is well-known, mesenchymal stem cells (MSCs) are a type of pluripotent stem cell that can differentiate into mesenchymal tissue lineage including osteoblast and adipocyte, the promotion of osteogenesis inhibits adipogenesis and vice versa (Lorthongpanich et al., 2019). During the OP process, the balance between osteogenesis adipogenesis is disturbed and the latter occupies an advantage. The ratio of fat in bone marrow leads to the reduction of osteoblasts, osteoclasts are activated afterward and finally causes the loss of bone mass (Uezumi et al., 2010; Chen et al., 2016; Hu et al., 2018; Liu et al., 2020). There are multitudinous signaling pathways and factors involved in this complex process, there into, the canonical Wnt/β-catenin signaling deserves attention. Research has indicated that β-catenin signaling regulates the differentiation of MSCs as a switch directly (Qiang et al., 2012). To be specific, once the β-catenin was inhibited, the osteogenesis of MSCs was restricted subsequently and the adipogenesis was enhanced on the contrary (de Winter and Nusse, 2021). Therefore, targeting β-catenin could be a potential strategy for OP treatment.

In the current study, we screened the optimal fraction of SSTZF in mice, furthermore, the mechanism of the anti-osteoporotic effect of SSTZF extract was elucidated by transgenic mice and cell experiments, *in vivo* and *in vitro* respectively.

## 2 Methods and Materials

### 2.1 Preparation of SSTZF Extract

All ten botanical drugs in SSTZF (Table 1) were provided by the First Affiliated Hospital of Zhejiang Chinese Medical University (Hangzhou China). The process of SSTZF extract preparation includes two parts: a concentrate of SSTZF preparation and secondary extraction with the organic solvent. The specific steps are as follows:

**TABLE 1:** Composition of SSTZ formula.

Chinese name	Botanical name	Family	Parts used	Weight (kg)
Hong hua	Carthamus tinctorius L.	Compositae	Flower	1.5
Di huang	Rehmannia glutinosa (Gaertn.) DC.	Plantaginaceae	Root	4.5
Du Zhong	Eucommia ulmoides Oliver.	Eucommiaceae	Bark	3
Fu zi	Aconitum carmichaelii Debeaux	Ranunculaceae	Root	3
Gou qi	Lycium barbarum L.	Solanaceae	Bark	3
Shan zhu yu	Cornus officinalis Siebold & Zucc.	Cornaceae	Fruit	1.5
Shan yao	Dioscorea oppositifolia L.	Dioscoreaceae	Fruit	3
Gan cao	Glycyrrhiza glabra L.	Leguminosae	Root	1.5
Tao ren	Prunus davidiana (CarriŠre) Franch.	Rosaceae	Seed	3
Rou gui	Cinnamomum cassia (L.) J.Presl	Lauraceae	Bark	1.5

After soaking in 6 volumes of distilled water for 1 h, CTL, RG, EUO, ACD, LBL, COS, DOL, GGL were mixed (the total dry weight was 21 kg) and in the ratio of 1:3:2:2:2:1:2:1(w/w) for reflux extraction (three times,1.5 h/time). PD and CCL were soaked in 5 volumes 60% EtOH for 1 h and were mixed in 2:1(w/w) (the total dry weight was 4.5 kg) for reflux extraction (three times, 1.5 h/time). Then the two portions of extracts were completely mixed into 7.5 L solution and then concentrated into the form of concentrated solution (3.4 g crude drug/mL). The procedures mentioned above are the preparation method of SSTZF concentrates. To extract its optimal anti-osteoporosis fraction, organic solvent including petroleum ether, ethyl acetate, and n-butanol solutions were used for further separation and extraction according to molecular polarity. 95 ml of the extract was diluted in 300 ml of distilled water, then, 300 ml of petroleum ether, 300 ml of ethyl acetate, and 300 ml of n-butanol solution were added and mixed in equal proportions respectively. The mixed solution was poured into the separating funnel and allowed to stand at room temperature for 12 h. The precipitation of the emulsion layer was discarded and the supernatant was collected. Each mixed solution was extracted three times in this mode. A rotary evaporator was used to volatilize the organic solvent completely, then the refined extract powders (2.8 g for petroleum ether extract, 10.3 g for ethyl acetate extract, 15.4 g for normal butanol extract) were obtained (Table 2). The powders were redissolved in 300 ml distilled water respectively and stored at −20°C. Each extract fraction was named SSTZF- petroleum ether extract (SSTZF-PE), SSTZF-ethyl acetate extract (SSTZF-EA), and SSTZF-n-butanol extract (SSTZF-NB).

**TABLE 2:** Fractions of SSTZF and yield.

Fraction	Isolated solvent	Isolated content (g)	Percentage of total crude extract (%)
SSTZF-PE	Petroleum ether	2.8	2.9
SSTZF-EA	Ethyl acetate	10.3	10.8
SSTZF-NB	Normal butanol	15.4	16.2

### 2.2 Experimental Animals

To construct the OVX mice model, 10 week-old female C57BL/6 mice were purchased from the Experimental Animal Center of Zhejiang Chinese Medical University (Hangzhou, China). For the sake of constructing growth plate chondrocytes -specific *β-catenin* conditional knockout mice, *β-catenin* ^*flox/flox*^ mice were crossed with *Gli1-CreER* ^*T2*^ transgenic mice to generate *Gli1-CreER* ^*T2*^; *β-catenin* ^*flox/flox*^ mice hereinafter referred to as *β-catenin* ^*Gli1ER*^ mice (Table 3). All original mice were purchased from Jackson Lab (Bar Harbor, ME, United States). To avoid gender-dependent differences, only females were selected for further experiments. To induce conditional gene knockout, tamoxifen was injected for three consecutive days (1 mg/10 g body weight, one time a day, intra-peritonelly) in 1-month-old mice. All studies were approved by the Animal Ethics Committee of Zhejiang Chinese Medical University (LZ12H27001).

**TABLE 3:** Breeding of *Gli1-CreER; β-catenin* ^*fx/fx*^ mice.

Breeding	Desired progeny
(a) Gli1-CreER × β-catenin^fx/fx^	(a) Gli1-CreER; β-catenin^fx/wt^
(b) Gli1-CreER; β-cateninfx/wt ×β-catenin^fx/fx^	(b) Gli1-CreER; β-catenin^fx/fx^
(c) Gli1-CreER; β-cateninfx/fx ×β-catenin^fx/fx^	(c) Gli1-CreER; β-cateninfx/fx andβ-catenin^fx/fx^

### 2.3 Experimental Groups and Drug Administration

All C57BL/6 mice were arranged into six groups (*n* = 6 in each group) randomly: the sham group, OVX group, SSTZF-PE group, SSTZF-EA group, SSTZF-NB group, and SSTZF group. According to the screening results, the transgenic mice were divided into three groups (*n* = 6 in each group): Cre-negative group, *β-catenin* ^*Gli1ER*^ group, and *β-catenin* ^*Gli1ER*^ + SSTZF-NB group. Both ovaries were removed in all C56BL/6 mice groups except the sham group. Instead, a sham operation that only excised the surrounding fat tissues equally and preserved bilateral ovaries intact was performed. SSTZF-PE, SSTZF-EA, SSTZF-NB were orally administrated to each corresponding group respectively for 10 consecutive weeks (0.2 ml/10 g body weight, once a day) from the day after OVX surgery. After tamoxifen inducement, SSTZF-NB were orally administrated to mice in the *β-catenin* ^*Gli1ER*^ + SSTZF-NB group. Other groups were treated with an identical dosage of PBS.

### 2.4 μCT Analyses

Samples of the femur from each group were collected and scanned with micro-CT (μCT). The cross-section of the distal femur metaphysis was reconstructed in three dimensions. Bone mineral density (BMD, g/mm^3^), bone volume fraction (BV/TV, %), average trabecular thickness (Tb.Th, mm), the average number of trabecular (1/mm), and average trabecular separation (Tb.Sp, mm) were collected for morphometry quantitative analysis.

### 2.5 Biomechanical Testing

To test the modulus of elasticity (MOE) and maximum loading of femurs in six groups of wild type mice, three-point bending test was performed with the use of the Axial-Torsion Fatigue Testing System (Instron, 5569R1412, United States).

### 2.6 Histology, Histomorphometry, and Immunohistochemistry

The preparation for the production of sections was processed as described previously (Xu et al., 2020). Then 3 μm sections at the site of femur metaphysis were cut coronally for Alcian Blue Hematoxylin/Orange G (ABH) staining. The indexes of histomorphometry including the area of lipid droplets and area of trabecular bone were detected with the use of OsteoMetrics software (Decatur, GA). IHC was performed using anti-alkaline phosphatase (ALP, ARIGO, ARG57422 1:300), anti-fatty acid-binding protein (FABP4, Abcam, ab92501, 1:200), anti-β-catenin (HuaBio, ER0805, 1:200). The quantitative analyses for positive staining area were performed by the software of image-pro plus6.0 (Media Cybernetics, Silver Spring, United States).

### 2.7 Cell Culture

Mesenchymal stem cells line C3H10T1/2 cells (ATCC, Manassas, VA, United States) were cultured in Alpha modified Eagle’s medium (Gibco, MD, United States) containing 10% (v/v) fetal bovine serum (FBS) (Sigma, MO, United States) and 1% penicillin and streptomycin (Gibco) at 37°C in 5% CO_2_ atmosphere.10%FBS, FBS containing 10 μg/ml SSTZF-NB and FBS containing 50 μg/ml SSTZF-NB were added to corresponding wells separately. With treatment for 72 h, cells were measured for qRT-PCR and western blot analyzes.

### 2.8 Real-Time Quantitative PCR

Total RNA was extracted from C_3_H_10_T1/2 cells with the use of TRIzol reagent (Invitrogen, CA, United States). Subsequently, 2 μg of total RNA was sucked out and used for cDNA synthesis by RevertAid First Strand cDNA Synthesis Kit (Invitrogen, CA, United States) following the manufacturer’s instructions. Then, the expression level of *ALP* and *FABP4* was determined by real-time quantitative PCR [relative to β-actin control with a QuantStudio™ 7 Flex Real-Time PCR System (Thermo Scientific, MA, United States)]. Forward and reverse sequence of the target gene are as follows: primer sequences of ALP, forward: 5′-TCC​TGA​CCA​AAA​ACC​TCA​AAG​G-3′, reverse: 5′-TCG​TTC​ATG​CAG​AGC​CTG​C-3′; sequences of FABP4, forward: 5′-AAA​TCA​CCG​CAG​ACG​ACA​GG-3′, reverse: 5′-GGC​TCA​TGC​CCT​TTC​ATA​AAC-3′; sequence of *β*-actin, forward: 5′-GGA​GAT​TAC​TGC​CCT​GGC​TCC​TA-3′, reverse: 5′-GAC​TCA​TCG​TAC​TCC​TGC​TTG​CTG-3′.

### 2.9 Western Blot

Total proteins were extracted respectively from C_3_H_10_T1/2 cells, which were cultured in control serum and serum containing SSTZF-NB, by using lysis buffer containing protease and phosphatase inhibitors, then incubated on ice for 30 min and isolated on a 12% SDS-PAGE gel. Samples were transferred on polyvinylidene fluoride membranes and blocked in 5% milk for 1.5 h. The membranes were incubated overnight at 4°C with anti-β-actin (1:5,000, Abcam, United Kingdom) and anti-β-catenin (1:1,000 HuaBio, CN). TBS-T was used to wash the membranes, and then membranes were incubated for 1 h with goat anti-rabbit horseradish peroxidase-conjugated secondary antibody (1:5,000, Abcam, MA, United States). The protein bands were visualized with Image Quant LAS 4000 (EG, United States). Finally, ImageJ was used to calculate the densitometry of each band for quantification.

### 2.10 UPLC/MS Analysis of SSTZF

Sample preparation for the Ultra Performance Liquid Chromatography (UPLC) analysis was as follows: 1 mg of SSTZF-NB was diluted in methanol/water (50/50) solution. Then, 1 ml of the previous liquid was diluted with methanol to 1/10 concentration. The test sample was harvested after being filtered with 0.22 μm membrane. The conditions of UPLC were as follows: the type of ACQUITY UPLC™ HSS T3 (100 mm × 2.1 mm, 1.8 μm) chromatographic column was selected. The sample size for injection, rate of flow, and column temperature were 1 μl, 0.3 ml/min, and 30°C respectively. The mobile phase was acetonitrile (A)—0.1% formic acid (B) and the elution gradients were as below: 00 min ∼ 7 min, 15%A–85%B; 7 ∼ 15 min, 35%A–65%B; 15–18 min, 60%A–40%B; 18 ∼ 23 min, 90%A–10%B; 23 ∼ 26 min, 90%A–10%B; 27–29 min, 5%A–95%B.

For Mass Spectrometry (MS), MS^E^ continuum mode was selected and the following parameter settings were used: ESI + mode: capillary voltage 3.0 kV; sample cone 40 V; source offset 80 V; source temperature 120°C; desolvation temperature 400°C; cone gas 50 L/h; desolvation gas 800 L/h; nebulizer 6.0Bar. TOF MS and TOF MS/MS were scanned with the mass range of m/z 50–1,500 and 50–1,500, respectively. 100 ng/ml leucine enkephalin solution was used as the calibration standard solution for quality control, and sodium-formate was used to calibrate the instrument. Accurate mass and composition for the precursor and fragment ions were analyzed by using UNIFI software integrated with the instrument. Base peak ion chromatogram of SSTZF-NB is shown in Supplementary File S1. The detected compounds are listed in Supplementary File S2.

### 2.11 Statistical Analysis

All data were presented as mean ± standard deviation. Student’s t**-**test and one-way ANOVA test followed by the Tukey-Kramer test were performed using SPSS 24.0 software. *p < 0.05 was considered statistically significant.

## 3 Results

### 3.1 Screening the Optimal Anti-osteoporotic Fraction of SSTZF Extract

**FIGURE 1:**
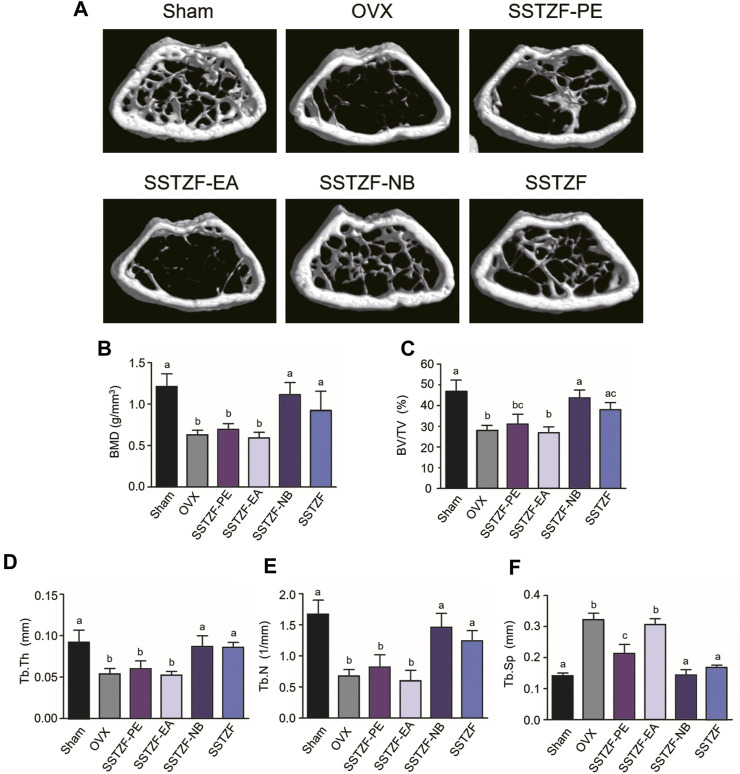
SSTZF-NB prevents bone loss in OVX mice. **(A)** Representative μCT images of each group. Quantification of microstructural data including BMD **(B)**, BV/TV **(C)**, Tb.Th **(D)**, Tb.N **(E)**, and Tb.Sp **(F)**. a–d: The means not sharing a common letter are significantly different among the groups at *p* < 0.05 by one-way ANOVA with Duncan’s multiple-range test.

To determine the optimal anti-osteoporotic fraction of SSTZF extract, three kinds of extracts and original SSTZF were orally administrated to the OVX mice respectively. No adverse events happened during the experiment. The 3D reconstruction images revealed that OVX could induce obvious bone loss of the distal femur metaphysis area when compared to sham group mice. SSTZF-PE and the original SSTZF could partially restrain deterioration, however, SSTZF-EA hardly works. Interestingly, SSTZF-NB showed a remarkable inhibitory effect on bone loss when compared to each intervention group (Figure 1A). The μCT analysis showed a similar trend. The indexes of BMD, BV/TV, Tb.Th, Tb.N, and Tb.Sp were significantly deteriorated after OVX inducement and significantly ameliorated by SSTZF-NB treatment (Figures 1B–F).

**FIGURE 2:**
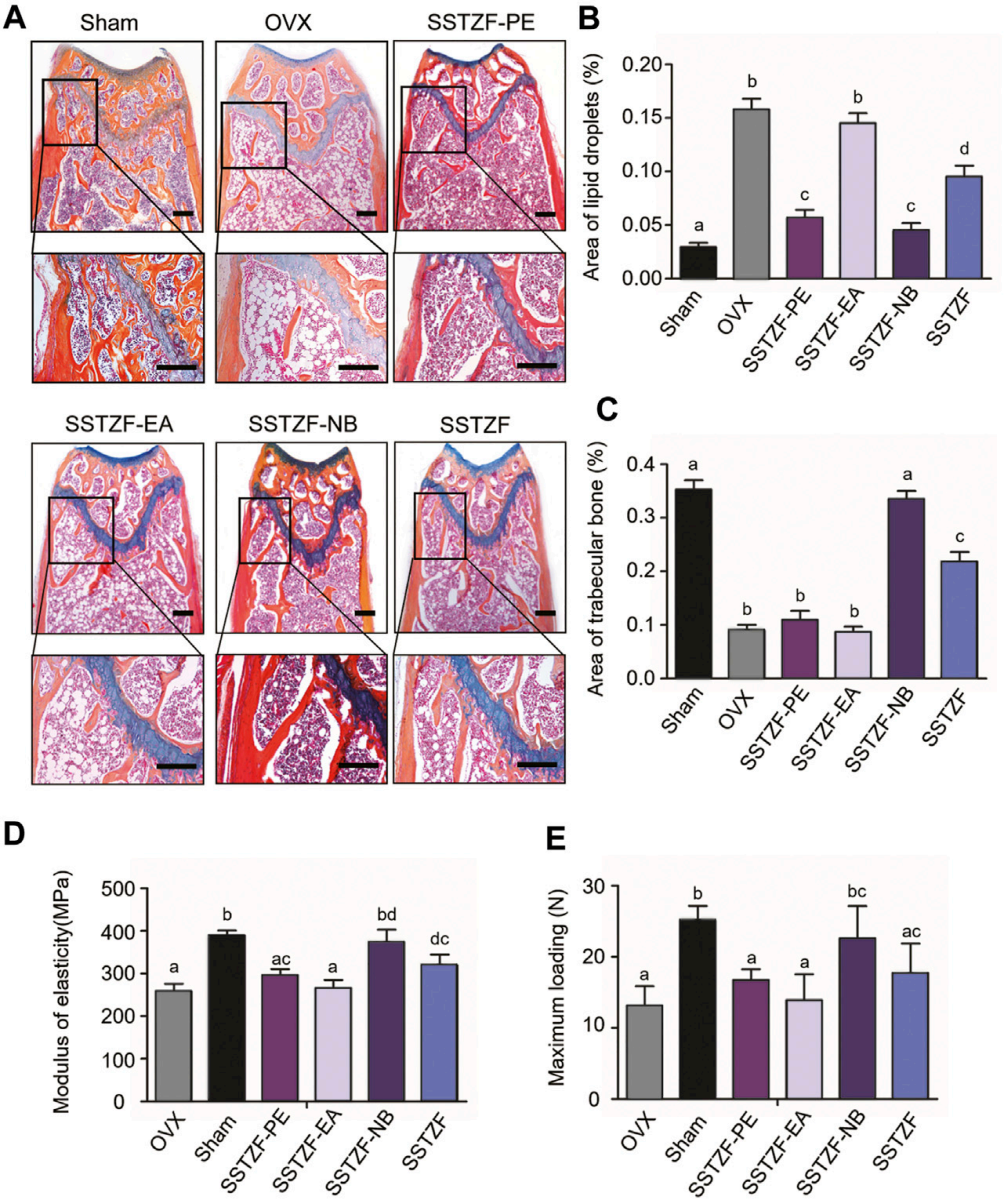
SSTZF-NB increases trabecular bone formation and decreases fat accumulation in the area of the chondro-osseous junction. **(A)** Alcian Blue Hematoxylin/Orange G staining of the distal femur. **(B)** The area of lipid droplets. **(C)** The area of trabecular bone. SSTZF-NB enhances bone strength. **(D)** The modulus of elasticity. **(E)** The max-loading. Scale bars: 1000 μm **A–D**: The means not sharing a common letter are significantly different among the groups at *p* < 0.05 by one-way ANOVA with Duncan’s multiple-range test.

Results of ABH staining and histomorphometry analyses showed that OVX surgery could cause serious trabeculae deterioration and fat droplet accumulation in the area of the femoral metaphysis, compared to the sham group. However, the lesion could be alleviated with the treatment of SSTZF-NB, but not with other extract fractions. SSTZF could partially improve the deterioration (Figures 2A–C).

Outcomes of biomechanical experiments indicated that OVX could lead to a decreased MOE and reduced max-loading when compared with the sham group, and these indexes were improved only in the SSTZF-NB group (Figures 2D–E). All data mentioned above testify that SSTZF-NB is the optimal anti-bone-loss fraction of SSTZF extract.

### 3.2 SSTZF-NB Fraction Upregulated the Expression of β-Catenin

**FIGURE 3:**
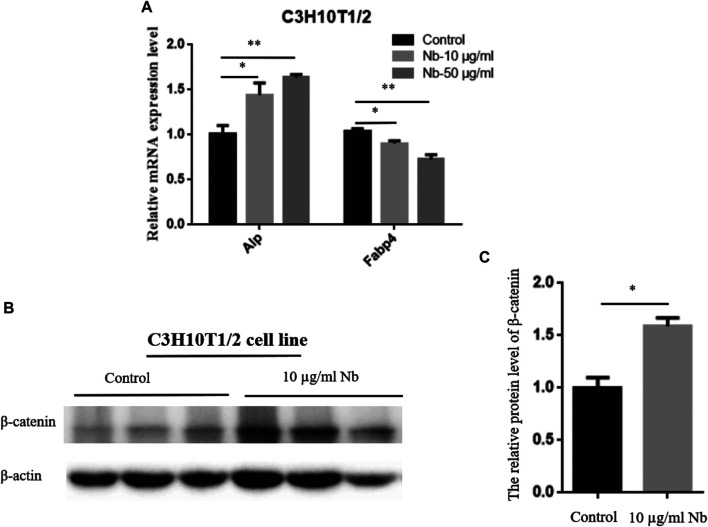
SSTZF-NB upregulates ALP mRNA, FABP4 mRNA expression **(A)**, and β-catenin protein level **(B)** in C3H10T1/2 cells. **(C)** The relative protein level of β-catenin.

To determine the possible molecular mechanism of SSTZF-NB anti-osteoporosis, qRT-PCR was performed to examine the effect of SSTZF-NB on the regulation of ALP and FABP4 mRNA expression in C_3_H_10_T1/2 cells. Surprisingly, we found that ALP was highly expressed while FABP4 was downregulated in C_3_H_10T_1/2 cells with any dose of SSTZF-NB treatment (Figure 3A). To provide more evidence of SSTZF-NB preventing bone loss, western bolt was performed to examine the protein expression level of *β-catenin* after being treated with SSTZF-NB. As expected, *β-catenin* was significantly upregulated in the low dose SSTZF-NB treatment group (Figures 3B,C). These data indicate SSTZF-NB anti-osteoporosis possibly via enhancing osteogenesis and inhibiting adipogenesis and are related to the activation of *β*-catenin.

### 3.3 SSTZF-NB Fraction Enhanced Osteogenesis and Inhibited Adipogenesis in OVX Mice

**FIGURE 4:**
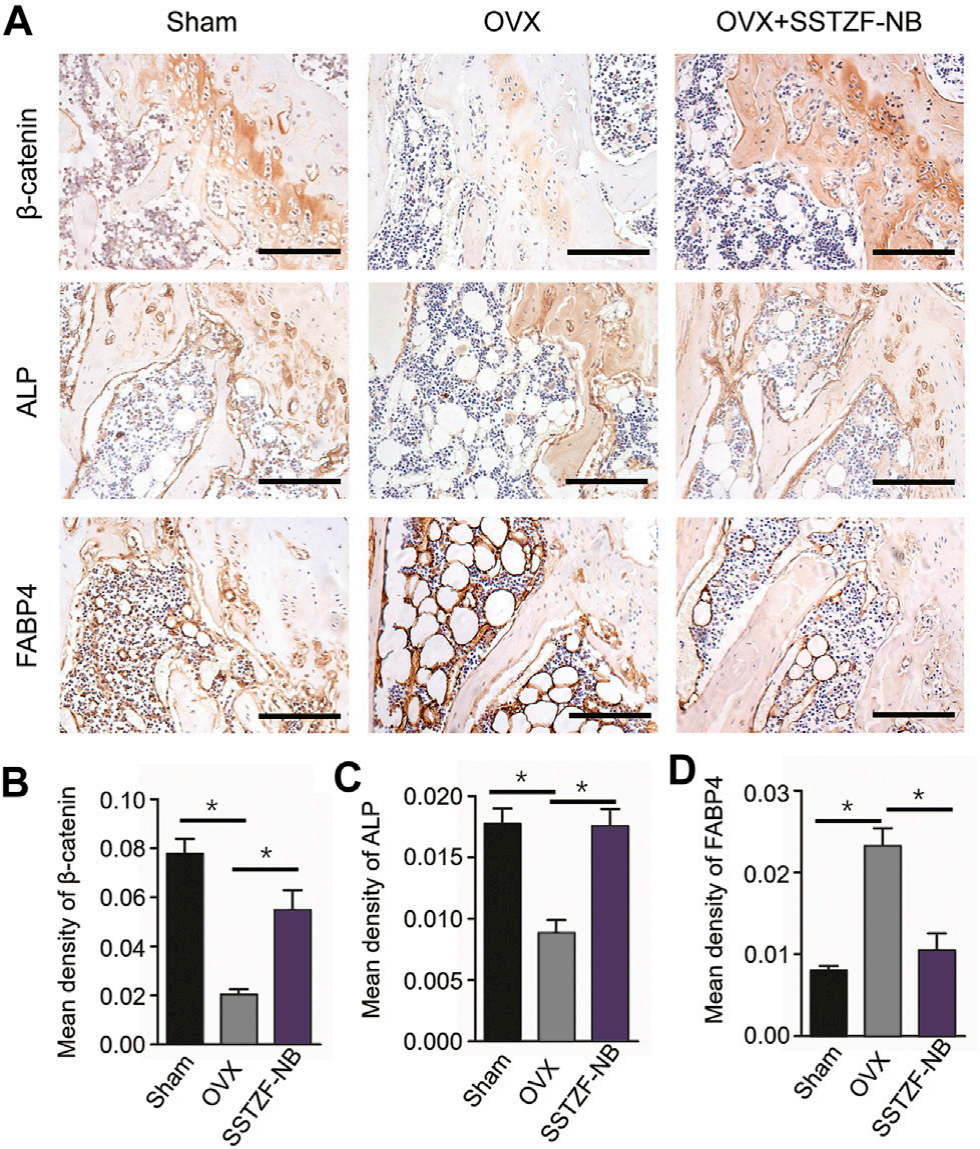
SSTZF-NB enhances osteogenesis and inhibits adipogenesis via activating β-catenin in mice. **(A)** Representative IHC staining images of β-catenin in growth plate chondrocytes, ALP, and FABP4 in the area of the chondro-osseous junction. Semi-quantitative analysis of positive expression of β-catenin **(B)**, ALP **(C)** and FABP4. **(D)** Scale bars: 1,000 μm *p < 0.001.

To further determine the molecular mechanism of the SSTZF-NB anti-osteoporotic IHC of ALP (an osteogenic specific matrix protein), FABP4 (a fatty acid-binding protein that specifically labels adipocytes) and *β-catenin* were used. Compared to the sham group mice, the expression of ALP was significantly downregulated while FABP4 was upregulated in the area of the chondro-osseous junction in the OVX mice. These phenomena were significantly reversed and ameliorated in the SSTZF-NB treatment group (Figures 4A–D). Furthermore, the expression level of *β-catenin* in growth plate chondrocytes was decreased in OVX mice and the reduction was elevated in SSTZF-NB group mice (Figures 4A,B).

### 3.4 SSTZF-NB Fraction Failed to Ameliorate Bone Loss and Lipid Drops Accumulation in *β*-catenin^Gli1ER^ Mice

**FIGURE 5:**
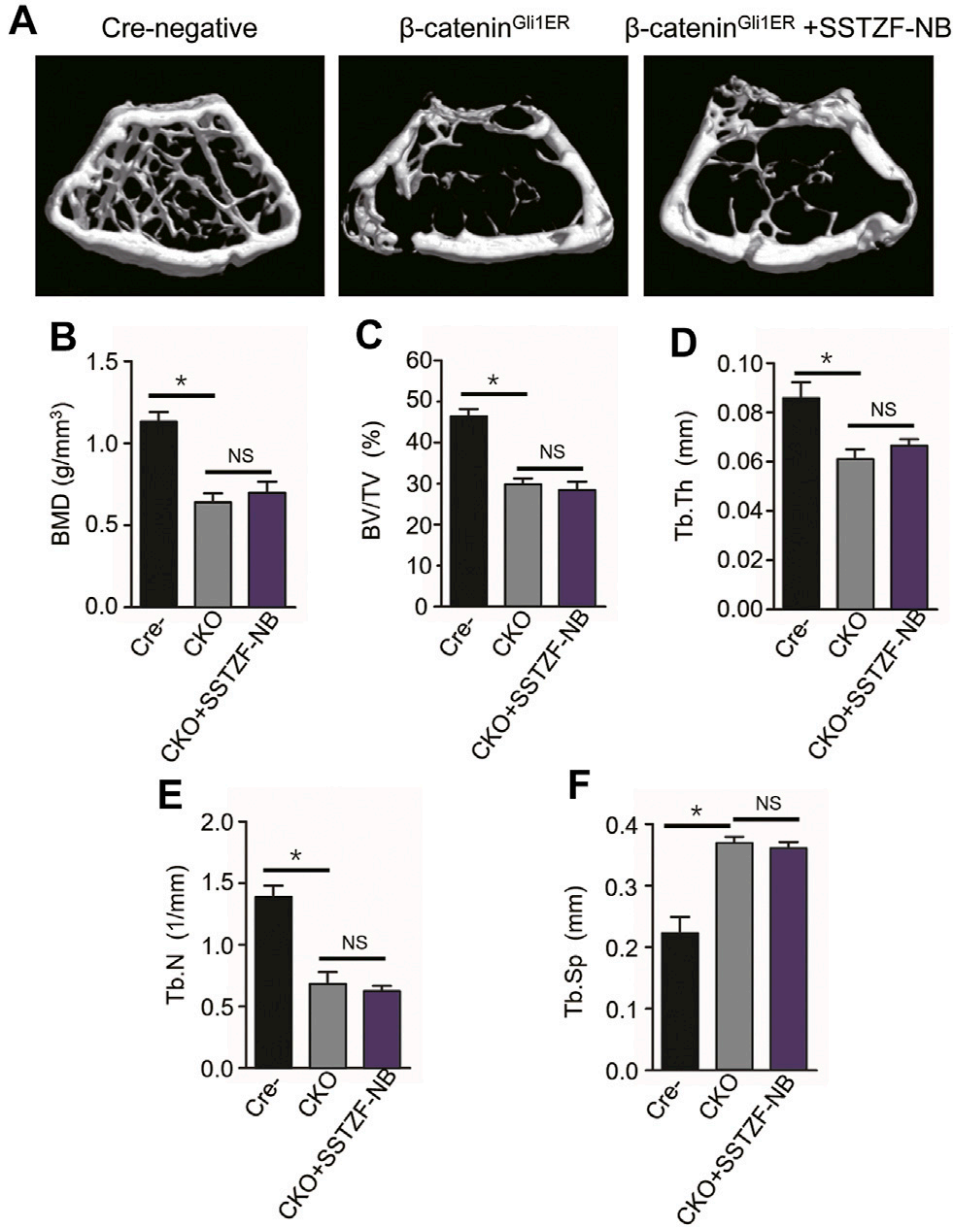
The positive effect of SSTZF-NB preventing bone loss is blocked in *β-catenin* ^*Gli1ER*^ mice. **(A)** Representative μCT image of each group. Quantification of microstructural data including BMD **(B)**, BV/TV **(C)**, Tb.Th **(D)**, Tb.N **(E)**, and Tb.Sp **(F)**. Cre-: Cre-negative mice group, CKO: *β-catenin* ^*Gli1ER*^ mice group, CKO + SSTZF-NB: *β-catenin* ^*Gli1ER*^ mice treated with SSTZF-NB group. NS: no significant difference. *p < 0.001.

**FIGURE 6:**
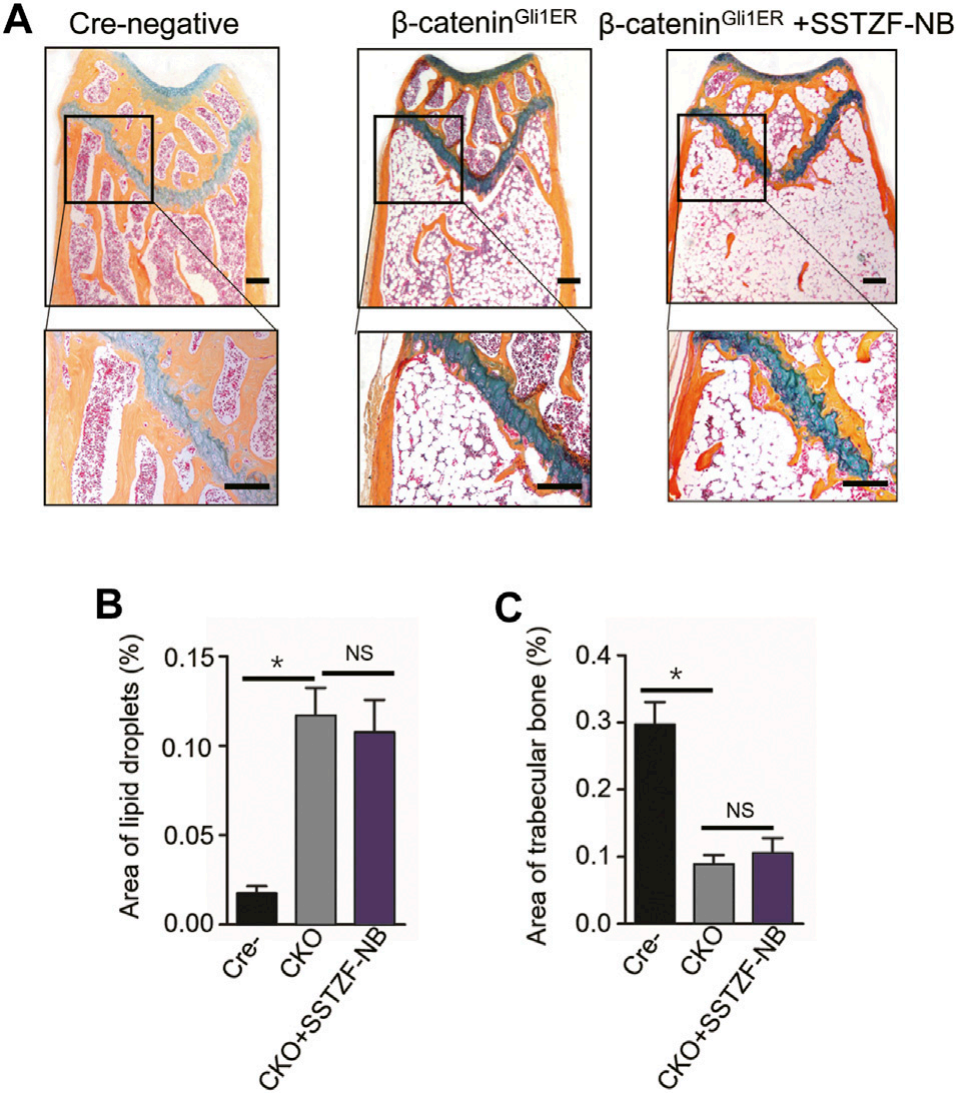
Influence of SSTZF-NB on increases of trabecular bone formation and decreases of fat accumulation in the area of the chondro-osseous junction inhibited in *β-catenin* ^*Gli1ER*^ mice. **(A)** ABH staining of each group. Scale bar: 1000 μm. Morphologic quantitative analyses of **(B)** lipid droplet area and **(C)** trabecular bone area. NS: no significant difference. *p < 0.001.

To provide conclusive evidence about the role of *β-catenin* signaling in SSTZF-NB preventing bone loss, growth plate-specific *β-catenin* conditional KO mice (*β-catenin* ^*Gli1ER*^) were generated. To examine the anti-osteoporotic effect of SSTZF-NB, μCT analyzes were first performed. Significantly bone loss was observed in *β-catenin* ^*Gli1ER*^ group mice as expected through the 3D constructed images when compared with Cre-negative group mice. Contrary to previous results, SSTZF-NB failed to alleviate the abnormality (Figure 5A). The indexes of BMD, BV/TV, Tb.Th, Tb.N, and Tb.Sp were not improved significantly in the *β-catenin* ^*Gli1ER*^ + SSTZF-NB group (Figures 5B–F). Additionally, according to the results of ABH staining, significant bone loss and lipid droplet accumulation at the area of femoral metaphysis were found in *β-catenin* ^*Gli1ER*^ group mice compared to cre-negative mice, especially in the chondro-osseous junction area. These pathological changes were also observed in *β-catenin* ^*Gli1ER*^ + SSTZF-NB group mice according to histologic and histomorphometric analyses (Figures 6A–C). These data indicate that SSTZF-NB could not ameliorate the osteoporosis-like changes caused by *β-catenin*-*deficiency.*

**FIGURE 7:**
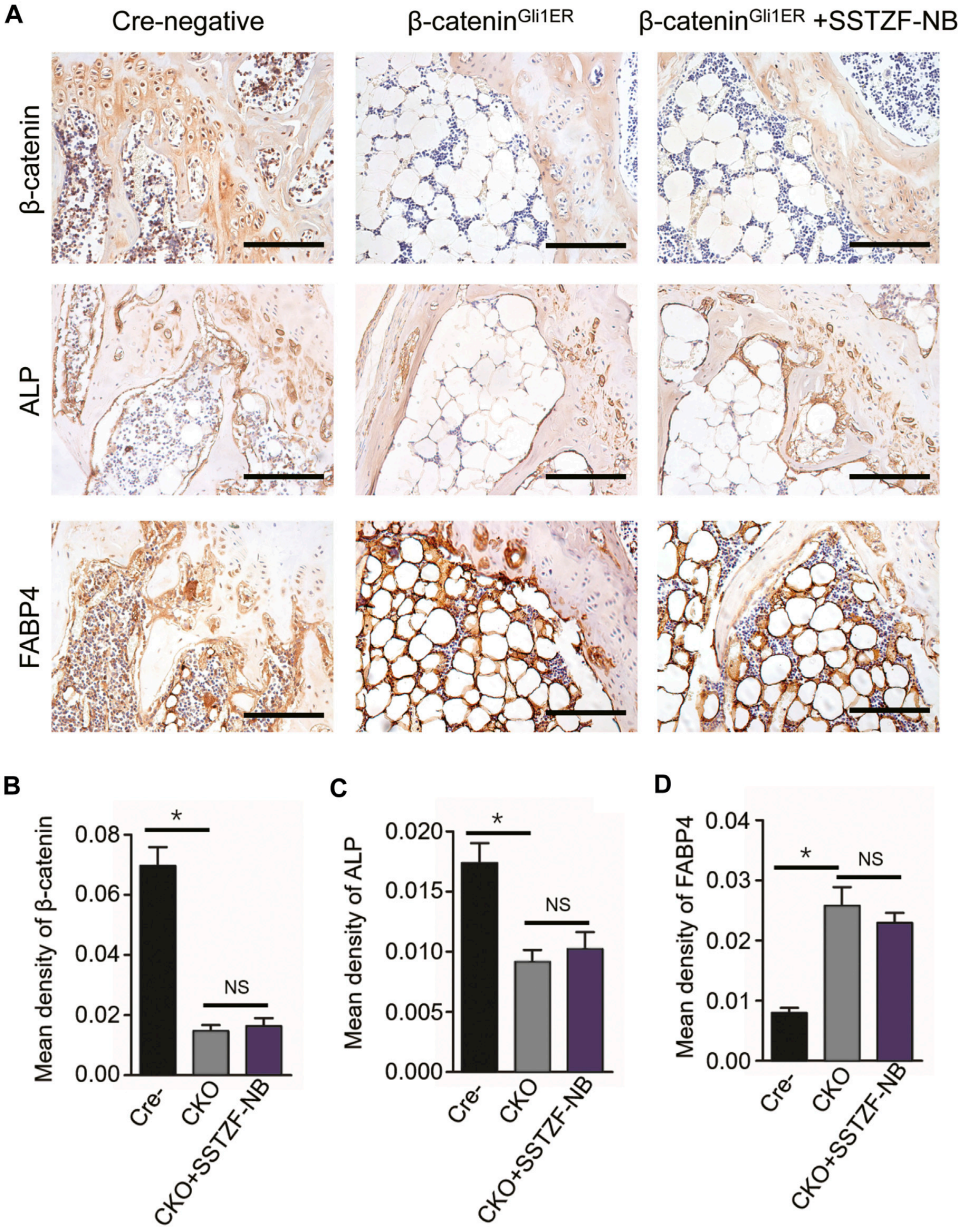
SSTZF-NB failure to restore the down-regulation of ALP and the up-regulation of FABP4 in *β-catenin* ^*Gli1ER*^ mice. **(A)** Representative IHC staining images of β-catenin in growth plate chondrocytes, ALP, and FABP4 in the area of chondro-osseous junction. Scale bars: 1,000 μm. Semi-quantitative analysis of positive expression of β-catenin **(B)**, ALP **(C)** and FABP4 **(D)**. *p < 0.001.

IHC assay showed that the protein expression of *β-catenin* decreased significantly in the area of the growth plate chondrocytes in *β-catenin* ^*Gli1ER*^ group mice when contrasted with cre-negative group mice. Furthermore, a decreased protein expression of ALP and an increased protein expression of FABP4, which could lead to osteoporotic-like changes, were also observed in the area of the chondro-osseous junction in *β-catenin* ^*Gli1ER*^ group mice. By contrast, these aberrant osteogeneses (decreased ALP) and adipogenesis (increased FABP4) induced by the inhibition of *β-catenin* signaling in growth-plate-chondrocytes were not restored by SSTZF-NB. No significant difference in the protein expression of ALP and FABP4 was found in *β-catenin* ^*Gli1ER*^ + SSTZF-NB group mice when compared with *β-catenin* ^*Gli1ER*^ group mice (Figures 7A–D).

## 4 Discussion

In this study, we confirmed that SSTZF-NB could ameliorate the osteoporotic-like phenotype and analyzed its potential mechanisms in mice models. Both μCT and histological results demonstrated that SSTZF-NB could prevent bone loss and alleviate fat accumulation. The fact that the positive effect of SSTZF-NB on anti-osteoporotic could be blocked when *β-catenin* was specifically deleted in growth plate chondrocyte which has been reported to have multi-differentiation potential, indicating that the mechanism of SSTZF-NB in preventing bone loss may largely depend on its contribution to the activation of *β-catenin* signaling in growth plate chondrocyte.

TCM is a popular and effective therapeutic method for treating osteoporosis. Shen-sui-tong-zhi formula is a classical anti-osteoporosis TCM that has a favorable curative effect in China. Although there have been many studies for the identification of the ten botanical drugs contained in SSTZ formula (Qu et al., 2016; Liang et al., 2019; Zheng et al., 2019; Wang et al., 2020), many phytochemicals exist in formula at the same time. Therefore, choosing the appropriate extraction method and combining the active ingredients is important for enhancing the curative effect and avoiding excessive intake of unnecessary chemical compounds (Wang et al., 2008). In this study, according to molecular polarity, petroleum ether, ethyl acetate, and n-butanol were selected as the extraction media for different fractions of the SSTZ formula. μCT analysis, biomechanical tests, and histomorphometry analysis consistently indicated that SSTZF-NB is the optimal anti-osteoporosis fraction. Then, UPLC/MS was used to identify its material basis. Unsurprisingly, a considerable number of the detected chemical components were reported to have the potential of promoting osteogenesis and anti-osteoporosis, like liquiritigenin, formononetin, etc. (Uchino et al., 2015; Mansoori et al., 2016; Gautam et al., 2017; Carnovali et al., 2020). In our study, the combination of these active components presented a satisfactory effect of preventing bone loss and restraining fat accumulation.

It is well known that *β-catenin* plays a key role in regulating osteogenic differentiation and adipogenic differentiation from MSCs. Moreover, the abnormality of osteogenesis and adipogenesis is a crucial part of the pathological progress of osteoporosis (Wu et al., 2018; Cao et al., 2019). Our research is consistent with previous studies, in which excessive fat accumulation and serious bone loss were observed in the area of chondro-osseous junction, additionally combined with a decreased protein expression of ALP and an increased expression of FABP4. Meanwhile, the expression of *β-catenin* was also downregulated in growth plate chondrocytes. Recent lineage tracing research indicates that Gli1-expressed growth plate chondrocytes have the properties of progenitor cells, which can differentiate into osteoblasts for osteogenesis, not only chondrogenesis (Shi et al., 2017; Haraguchi et al., 2018). Therefore, to further confirm whether SSTZF-NB could cause anti-osteoporosis when *β-catenin* signaling was inhibited in growth plate chondrocytes, *β-catenin* ^*Gli1ER*^ mice were generated and utilized. However, the positive effect of SSTZF-NB on anti-osteoporosis was almost blocked, neither osteogenesis nor adipogenesis, two of the most important aspects of osteoporosis, were ameliorated following SSTZF-NB administration in *β-catenin* ^*Gli1ER*^ mice. These results demonstrated that SSTZF-NB enhances osteogenesis and then acts as an anti-osteoporotic function mainly via activation of β-catenin signaling.

## 5 Conclusion

The data of this study illuminated the potentiation and molecular mechanism of SSTZF-NB in treating osteoporosis. Based on the aforementioned findings, we conclude that SSTZF-NB enhances osteogenesis mainly via activation of β-catenin signaling in growth plate chondrocytes. Our findings provide a salutary alternative preventive option for osteoporosis.

## Data Availability

The original contributions presented in the study are included in the article/Supplementary Material, further inquiries can be directed to the corresponding authors.
