# Supplementary material for: Fractions of Shen-Sui-Tong-Zhi Formula Enhance Osteogenesis Via Activation of β-Catenin Signaling in Growth Plate Chondrocytes
Source: Front Pharmacol. 2021 Sep 24;12:711004. doi: 10.3389/fphar.2021.711004 (PMC8498212; doi:10.3389/fphar.2021.711004)
Supplement: Supplementary file 1 [file DataSheet1.docx]

**Supplementary File 1.** **Base peak ion chromatogram of SSTZF-NB**


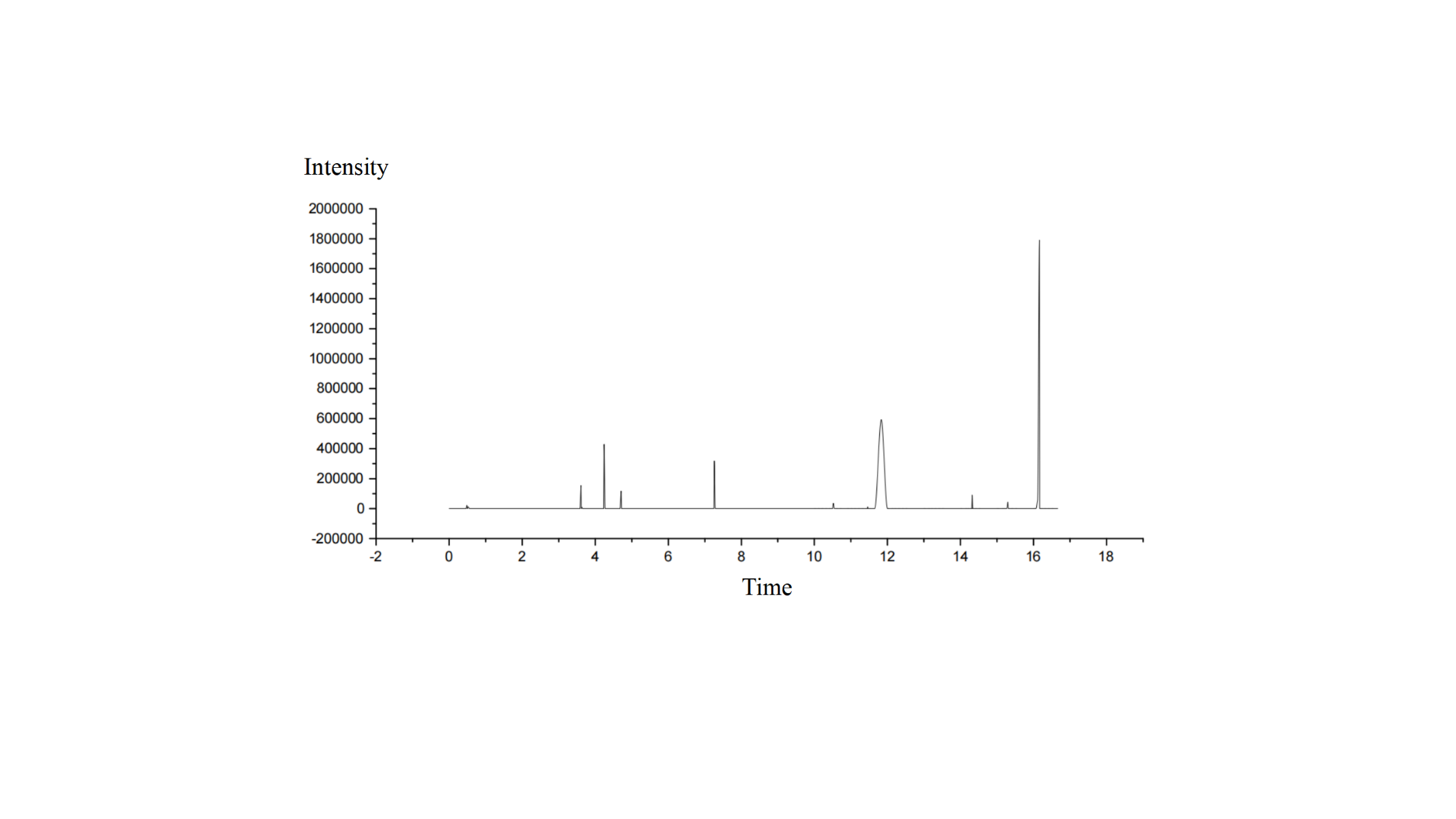


**Supplementary File 2. Identification of chemical components of SSTZF normal butanol extract by UPLC / MS**

| name | molecular formula | retention time（min） | response | ion peak  （m/s） | detected molecular mass (Da) | standard molecular mass (Da) |
| --- | --- | --- | --- | --- | --- | --- |
| Valine | C5H11NO2 | 0.49 | 21730 | 118.0855 | 117.0783 | 117.079 |
| Adenosine | C10H13N5O4 | 0.51 | 5218 | 268.104 | 267.0967 | 267.0968 |
| O-methoxycinnamaldehyde | C10H10O2 | 0.51 | 11847 | 180.1005 | 162.0667 | 162.0681 |
| Amygdalin | C20H27NO11 | 3.61 | 155069 | 475.1925 | 457.1587 | 457.1584 |
| Wild cherry bark glycosides | C14H17NO6 | 3.61 | 9131 | 296.1123 | 295.105 | 295.1056 |
| 15-α-hydroxy neokonitine | C24H39NO7 | 4.24 | 429141 | 454.2799 | 453.2726 | 453.2727 |
| Mourinho ning | C24H39NO6 | 4.71 | 115843 | 438.2851 | 437.2778 | 437.2777 |
| Liquiritigenin | C15H12O4 | 7.26 | 317641 | 257.0806 | 256.0733 | 256.0736 |
| Isoliquiritigenin | C15H12O4 | 10.51 | 35386 | 257.0803 | 256.073 | 256.0736 |
| Liquiritin | C21H22O9 | 10.51 | 31986 | 419.1335 | 418.1262 | 418.1264 |
| Coryneine | C11H17NO2 | 11.46 | 11355 | 234.089 | 195.1258 | 195.1259 |
| Salsolinol | C11H15NO2 | 11.46 | 8659 | 232.075 | 193.1119 | 193.1103 |
| Benzoylhypacoitine | C31H43NO9 | 11.83 | 593689 | 574.3014 | 573.2941 | 573.2938 |
| Isoglabrolide | C30H44O4 | 14.32 | 90243 | 469.3311 | 468.3238 | 468.324 |
| Glabrolide | C30H44O4 | 15.3 | 43437 | 469.3308 | 468.3235 | 468.324 |
| Enoxolone | C30H46O4 | 16.11 | 57215 | 471.3467 | 470.3394 | 470.3396 |
| Glycyrrhizic acid | C42H62O16 | 16.11 | 1788679 | 823.4116 | 822.4044 | 822.4038 |
